# Robust evidence for bisexual orientation among men

**DOI:** 10.1073/pnas.2003631117

**Published:** 2020-07-20

**Authors:** Jeremy Jabbour, Luke Holmes, David Sylva, Kevin J. Hsu, Theodore L. Semon, A. M. Rosenthal, Adam Safron, Erlend Slettevold, Tuesday M. Watts-Overall, Ritch C. Savin-Williams, John Sylla, Gerulf Rieger, J. Michael Bailey

**Affiliations:** ^a^Department of Psychology, Northwestern University, Evanston, IL 60208;; ^b^Department of Psychology, Essex University, Colchester CO4 3SQ, United Kingdom;; ^c^Department of Psychiatry, Kaiser Permanente, Los Angeles, CA 90056;; ^d^Department of Psychological and Social Sciences, Pennsylvania State University Abington, Abington, PA 19001;; ^e^Kinsey Institute, Indiana University, Bloomington, IN 47405;; ^f^School of Psychology, University of East London, Stratford E15 4LZ, United Kingdom;; ^g^Department of Psychology, Cornell University, Ithaca, NY 14853-4401;; ^h^American Institute of Bisexuality, Los Angeles, CA 90014;; ^i^University of Chicago Law School, University of Chicago, Chicago, IL 60637

**Keywords:** sexual orientation, bisexuality, sexual arousal, Kinsey scale, sexuality

## Abstract

The question whether some men have a bisexual orientation—that is, whether they are substantially sexually aroused and attracted to both sexes—has remained controversial among both scientists and laypersons. Skeptics believe that male sexual orientation can only be homosexual or heterosexual, and that bisexual identification reflects nonsexual concerns, such as a desire to deemphasize homosexuality. Although most bisexual-identified men report that they are attracted to both men and women, self-report data cannot refute these claims. Patterns of physiological (genital) arousal to male and female erotic stimuli can provide compelling evidence for male sexual orientation. (In contrast, most women provide similar physiological responses to male and female stimuli.) We investigated whether men who self-report bisexual feelings tend to produce bisexual arousal patterns. Prior studies of this issue have been small, used potentially invalid statistical tests, and produced inconsistent findings. We combined nearly all previously published data (from eight previous studies in the United States, United Kingdom, and Canada), yielding a sample of 474 to 588 men (depending on analysis). All participants were cisgender males. Highly robust results showed that bisexual-identified men’s genital and subjective arousal patterns were more bisexual than were those who identified as exclusively heterosexual or homosexual. These findings support the view that male sexual orientation contains a range, from heterosexuality, to bisexuality, to homosexuality.

## Significance

There has long been skepticism among both scientists and laypersons that male bisexual orientation exists. Skeptics have claimed that men who self-identify as bisexual are actually homosexual or heterosexual. (The existence of female bisexuality has been less controversial.) This controversy can be resolved using objective, genital responses of men to male and female erotic stimuli. We combined nearly all available data (from eight previous American, British, and Canadian studies) to form a dataset of more than 500 men, much larger than any previous individual study, and conducted rigorous statistical tests. Results provided compelling evidence that bisexual-identified men tend to show bisexual genital and subjective arousal patterns. Male sexual orientation is expressed on a continuum rather than dichotomously.

The status of male bisexuality as a sexual orientation—that is, the idea that some men are sexually aroused and attracted to both sexes—has a controversial history (1). Although some men identify as bisexual and have sexual experiences with men and women, the extent to which this reflects an underlying bisexual orientation has been questioned. Early sex researchers Krafft-Ebing (2) and Hirschfeld (3) believed that bisexual behavior and identification occurred primarily among monosexual (i.e., either heterosexual or homosexual) men for reasons other than a bisexual orientation. For example, some homosexual men identify as bisexual, or engage in sex with women, due to social pressures that favor heterosexuality. In response to those who doubted the existence of a bisexual orientation, Kinsey proposed a quasi-continuous scale of sexual orientation, proclaiming: “Males do not represent two discrete populations, heterosexual and homosexual. The world is not to be divided into sheep and goats. Not all things are black nor all things white” (ref. 4, pp. 638–639). With his scale, Kinsey demonstrated that self-reported bisexual attraction and behavior are not rare. However, because the scale relied on self-reports, results could not provide definitive evidence for bisexual orientation. For example, surveys have shown that a large proportion of men who identify as gay or homosexual had gone through a previous and transient phase of bisexual identification (5, 6).

Other reasons why bisexual men’s self-reported sexual feelings have sometimes been questioned likely include cognitive and emotional biases of the questioners. Some heterosexual and homosexual men may find it relatively easy to understand each other’s monosexuality because both have strong sexual attraction to one sex and virtually none to the other. For this reason, these men may have more difficulty accepting bisexuality as it challenges their binary conceptualizations of sexual orientation (7). Furthermore, bisexual individuals may be mistrusted and stigmatized by both heterosexual and homosexual people, and perceived as untrustworthy, promiscuous, and unable to commit (8–10).

Self-reported measures of sexual attraction, interest, and arousal are useful and ubiquitous in sex research. When self-reports are questioned, however, other valid measures are desirable. One promising approach to empirical verification of self-reported male bisexuality as an orientation uses penile plethysmography (i.e., a strain gauge around the penis) to study genital sexual arousal patterns to erotic stimuli featuring men or women (but not both). Examples of stimuli used in these studies include videos of sexual interactions between actors or of solitary actors masturbating (11, 12). Such an approach has several advantages: It relies on physiological processes rather than self-report; it is difficult to consciously manipulate (13); and, for men, sexual arousal to attractive women or men is arguably equivalent to sexual orientation (1). This approach has been used in a handful of studies focusing on male bisexuality with mixed results. Some studies failed to provide evidence that bisexual-identified men had bisexual arousal patterns (11, 14). One other study with stringent recruitment criteria (i.e., minimum criteria for both sexual and romantic experience across sexes) found evidence for bisexual arousal (12). A recent study using less stringent recruitment criteria also found evidence that bisexual-identified men had bisexual physiological arousal patterns (15). All existing studies have been of small to modest size; the largest had 114 participants. Notably, across these studies, bisexual-identified men self-reported subjective arousal to both male and female stimuli, even in samples where their genital arousal did not reflect such a pattern.

Previous research may have not employed sufficiently rigorous statistical tests, further complicating the question of whether bisexual-identified men show bisexual physiological arousal patterns. Crucial predictions regarding bisexual orientation concern U-shaped (or inverted U-shaped) distributions, which previous studies tested via quadratic regression. However, this test may be insufficient to reliably detect U-shaped distributions (16). This is because significant quadratic regressions can occur if a linear regression changes slope over the range of the predictor, even if the sign of the slope does not change. Demonstrating U-shaped distributions without the threat of incorrect interpretation requires showing slope sign reversal from low to high values of the predictor. For example, if the left arm of the estimated regression slope is significantly positive, then the other arm needs to be significantly negative in order to result in a valid, inverse U-shaped estimate.

With the limitations of previous work in mind, the aim of this study was to examine the extent to which men who self-report bisexual orientation exhibit bisexual genital and self-reported arousal patterns. Our study is unique with respect to its large sample and its employment of a version of Simonsohn’s (16) “two-lines” test of U-shaped (or inverted U-shaped) distributions. Data included 606 male participants (with 474 remaining for genital analyses and 588 remaining for self-reported analyses following exclusions) (*Materials and Methods*) from American, Canadian, and British studies that collected data on men’s self-reported Kinsey scores and their genital and self-reported arousal to male and female erotic stimuli and to neutral stimuli (e.g., footage of landscapes and wildlife). These studies were conducted over the course of approximately two decades, from the years 2000 to 2019. Kinsey scores range from 0 (exclusively heterosexual) to 3 (equal attraction to both sexes) to 6 (exclusively homosexual). Scores of 0 and 6 are usually considered monosexual, and 1 to 5 nonmonosexual. Scores of 2 to 4 are generally accepted to comprise the bisexual range of the Kinsey scale (17).

This study focuses only on male sexual orientation, despite the equal scientific importance of understanding female sexual orientation, for several related reasons. The question of whether bisexual arousal patterns exist has been less controversial about women than men (1). Historically, there was no parallel debate about female sexual orientation to that between skeptics [e.g., Krafft-Ebing (2) and Hirschfeld (3)] and proponents (e.g., Kinsey) (4) of the validity of male bisexuality. Recent scientific developments have supported important and potentially relevant differences in the expression of male and female sexual orientation. In laboratory research, the large majority of women exhibit similar subjective and physiological sexual arousal to both male and female stimuli, despite heterosexual identification (18, 19). Furthermore, the idea that female sexuality is especially “fluid” with respect to gender, with some women situationally attracted to men or women depending on circumstances, has been well-established (20). Male, but not female, self-reported sexual orientation shows a bimodal distribution (21), supporting the idea that male bisexuality is relatively uncommon whereas female bisexuality is less so. Thus, converging lines of evidence suggest that there are important differences in the expression of male and female sexual orientation, perhaps especially bisexuality. Consequently, research exploring the validity of bisexual identification–and especially research comparing the genital response of bisexual and monosexual persons–has been pursued more vigorously for male than for female sexual orientation. The men cumulatively studied in the research on male sexual orientation have been aggregated to comprise the large sample used in the present study.

## Results

**Fig. 1.**
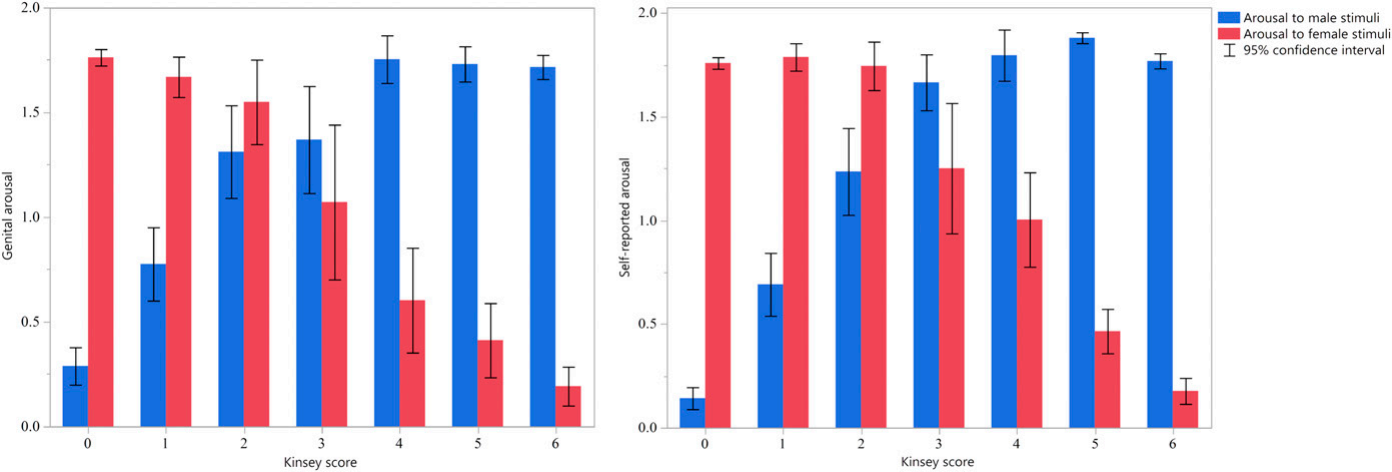
Mean standardized genital (*Left*) and self-reported (*Right*) arousal to female and male stimuli (±95% CI) for men of different Kinsey scores, after subtracting response to neutral stimuli. The *y* axis is measured in units of within-subjects z-scores.

Fig. 1 presents participants’ ipsatized (i.e., standardized within subjects across erotic and neutral stimuli) genital and self-reported arousal to female and male stimuli across the Kinsey scale, in within-subject SDs. Only participants who produced adequate arousal for our main analyses were included. The figure shows that the relative response to female and male stimuli closely tracked the Kinsey scale, on the whole. The difference in genital arousal to females minus males correlated strongly with the Kinsey scale (*r*[472] = 0.838, 95% CI [0.809, 0.863], *P* < 0.0001). The analogous correlation of self-reported arousal with the Kinsey scale was also strong (*r*[586] = 0.916, 95% CI [0.902, 0.928], *P* < 0.0001).

Exclusively heterosexual and homosexual men (who have Kinsey scores of 0 and 6, respectively) showed larger mean differences in their arousal to male and female stimuli compared with men who have intermediate Kinsey scores (i.e., scores of 1 to 5). Although this pattern is consistent with the possibility that intermediate Kinsey scores are associated with relatively bisexual arousal patterns, it is also consistent with an alternative explanation. It would be possible to create the mean arousal scores of men with Kinsey scores 1 to 5 (which appear relatively bisexual) by mixing men with arousal patterns similar to the means for Kinsey 0 (exclusively heterosexual) with those similar to Kinsey 6 (exclusively homosexual). Therefore, simply averaging each Kinsey group’s responses to male and to female stimuli can in principle produce misleading results. Thus, results depicted in Fig. 1 by themselves cannot provide conclusive evidence that men who report bisexual attractions have a more bisexual arousal pattern than monosexual men.

**Fig. 2.**
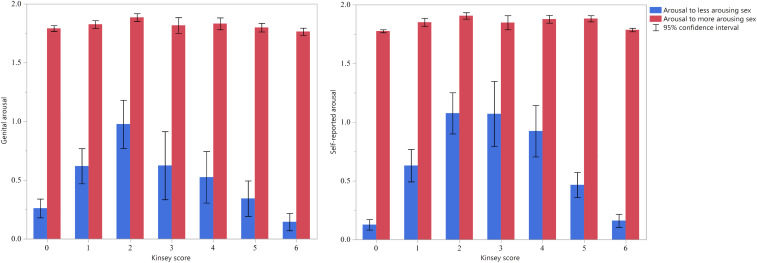
Mean standardized genital (*Left*) and self-reported (*Right*) arousal (±95% CI) to the more and less arousing sex for men of different Kinsey scores, after subtracting response to neutral stimuli. The *y* axis is measured in units of within-subjects z-scores. Values for arousal to the less arousing sex should show an inverted U-shaped distribution if men with Kinsey scores in the bisexual range show bisexual arousal patterns, and a flat distribution if they do not.

Two alternative analyses can provide more definitive evidence (11, 12). Both rely on variables depicted or derived from those in Fig. 2: responses to the more-arousing sex and responses to the less-arousing sex. These variables were determined empirically for each individual. Men have relatively bisexual arousal patterns if 1) their responses to their less arousing sex exceeds that of other men, and 2) the difference between their responses to their more and to their less arousing sex is less than that of other men.

The first criterion for bisexual arousal patterns is demonstrated by considering that men with a bisexual arousal pattern should show more arousal to male stimuli compared with heterosexual men and more arousal to female stimuli compared with homosexual men. Heterosexual men’s less-arousing sex will usually be “male” and homosexual men’s “female.” (Measurement error may prevent this generalization from always being true.) Thus, the first criterion is that bisexual men should show more arousal to erotic stimuli depicting their (empirically defined) less-arousing sex, compared with homosexual and heterosexual men. The second criterion is demonstrated by considering that men with a bisexual arousal pattern should show an especially small unsigned difference between their arousal to male and female stimuli, compared with heterosexual and homosexual men. This difference is equivalent to that between responses to the more arousing sex minus responses to the less arousing sex.

We henceforth refer to the two key dependent variables as Minimum Arousal (i.e., responses to the less arousing sex) and Absolute Arousal Difference (i.e., the unsigned value of the difference between arousal to female stimuli and arousal to male stimuli). The two dependent variables derived from Fig. 2 were almost perfectly negatively correlated with each other: for genital arousal, *r* = −0.976 and for self-reported arousal, *r* = −0.944. This strong correspondence is partly an artifact of standardizing within participants using only three scores (i.e., average arousal to male, to female, and to neutral stimuli), especially when two of the scores tend to be similar to each other and different from the third score. Because Minimum Arousal and the Absolute Arousal Difference are not generally so highly correlated (for example, for the unstandardized data we analyzed subsequently, their correlation for genital arousal was *r*[474] = −0.028), and because they are conceptually distinct, we have retained both variables in our main analyses.

In addition, we created a composite variable using Minimum Arousal and Absolute Arousal Difference, by standardizing both across participants, changing the sign of the Absolute Arousal Difference and then taking their average. We refer to this variable as the Bisexual Arousal Composite, and men with a relatively bisexual arousal pattern should have high scores on it. Although the composite was almost entirely redundant with Minimum Arousal and Absolute Arousal Difference—as the latter are with each other—for the ipsatized data, we retained all three variables because in some subsequent analyses using untransformed data, they were much less highly correlated.

If men who self-report Kinsey scores in the bisexual range indeed have relatively bisexual arousal patterns, then both Minimum Arousal and the Bisexual Arousal Composite should show an inverted U-shaped distribution across the Kinsey range (i.e., men who self-identify as 0 [exclusively heterosexual] and 6 [exclusively homosexual] should have the lowest scores for these variables; men in intermediate groups should have greater values, with the peak resting at a Kinsey score of 3); the Absolute Arousal Difference should show a U-shaped distribution (i.e., exclusively heterosexual and exclusively homosexual men should have lower values than bisexual-identified men). Conversely, if men who indicate that they are relatively bisexual have monosexual arousal patterns in actuality, then the values for these three variables should be evenly distributed across the Kinsey scale, and we should have a flat, horizontal line, rather than a U-shaped distribution. A rigorous demonstration that bisexual men have relatively bisexual arousal patterns requires a change of sign of regression slopes across the Kinsey scale. The method proposed by Simonsohn (16), the two-lines test, requires establishing that, for some break point on the predictor variable, if one conducts separate regression analyses using data on either side of the point, both regression slopes are statistically significant but of opposite sign.

**Fig. 3.**
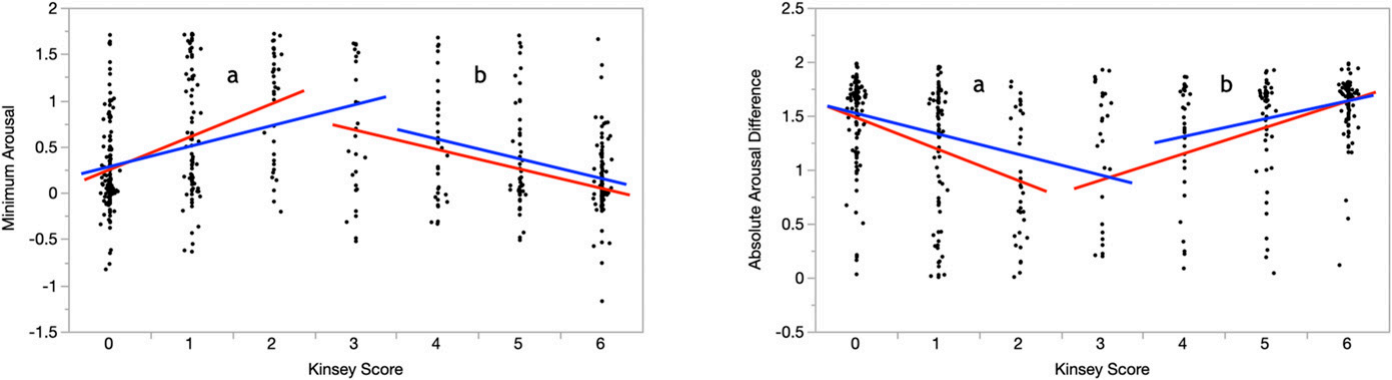
Regression lines whose slopes comprise the “two-lines” analysis for Standardized Minimum Genital Arousal (*Left*) and Standardized Absolute Genital Arousal Difference (*Right*). Evidence for bisexual orientation requires that Minimum Arousal have an inverted U-shaped distribution and that Absolute Arousal Difference be U-shaped. The alternative hypothesis is that values will be evenly distributed across the Kinsey scale. Each variable has four associated regression lines: two using a break point of Kinsey Score = 2.5 (in red) and two using a break point of Kinsey Score = 3.5 (in blue). (Analyses using both break points were conducted to evaluate robustness with respect to break point choice.) For each break point and variable, there were two crucial tests: the slope of the regression lines to the left (a) and to the right (b) of the break point should be consistent with the predicted distribution, should be of opposite sign, and should be statistically significant. These predictions were confirmed. (See Table 1.)

We modified this method as follows. Instead of using Simonsohn’s algorithm for locating one optimal break point, we conducted two sets of analyses using two different break points: 2.5 and 3.5. Our modification was motivated by both necessity and a desire to explore robustness. The middle of the Kinsey distribution is 3, and a Kinsey score of 3 signifies the greatest degree of bisexuality. As such, that score is the best guess for the inversion point of the hypothesized U-shaped and inverted U-shaped distributions. However, the Kinsey score 3 is unavailable as a break point because the break point should not include scores that actually exist in the data. The analysis with 2.5 as the break point compares the correlations between the Kinsey scores and the dependent variables in the range of Kinsey 0 to 2 with the respective correlations in the range of Kinsey 3 to 6. (Note that, because our Kinsey score variable includes only whole numbers, any break point between 2 and 3 is equivalent to a break point of 2.5; all provide exactly the same separation of points.) The analysis using the break point 3.5 compares the correlations in the Kinsey range 0 to 3 with those in the Kinsey range of 4 to 6. Examining results using two different break points in separate analyses allowed us to examine the robustness of results across them. Fig. 3 presents the regression lines comprising the two lines tests for both sets of break points, for both Standardized Minimum Genital Arousal (Fig. 3, *Left*) and Standardized Absolute Genital Arousal Difference (Fig. 3, *Right*).

Table 1 includes results of the two-lines analyses for both break points. For analyses of genital arousal, we included data from 474 men with sufficient genital responses. For analyses of self-reported arousal, we included data from 588 men who provided adequate self-reported arousal data. We present standardized correlations because the scale of the variables is more intuitively interpretable than unstandardized coefficients. All correlations were in directions consistent with more bisexual arousal tending to occur toward the middle of the Kinsey scale. The 95% CIs for all correlations excluded zero, usually by a large margin.

**Table 1.** Results of two-lines analyses for both break points

Break point	2.5				3.5			
	Slope for Kinsey 0 to 2		Slope for Kinsey 3 to 6		Slope for Kinsey 0 to 3		Slope for Kinsey 4 to 6	
	*r* (95% CI)	*P*	*r* (95% CI)	*P*	*r* (95% CI)	*P*	*r* 95% CI]	*P*
Genital analyses	(*n* = 253, df = 251)		(*n* = 221, df = 219)		(*n* = 279, df = 277)		(*n* = 195, df = 193)	
Minimum arousal	0.420 (0.312, 0.516)	<0.0001	−0.330 (−0.443, −0.207)	<0.0001	0.325 (0.216, 0.426)	<0.0001	−0.292 (−0.415, −0.158)	<0.0001
Absolute difference	−0.426 (−0.522, −0.320)	<0.0001	0.351 (0.230, 0.462)	<0.0001	−0.337 (−0.437, −0.228)	<0.0001	0.302 (0.169, 0.425)	<0.0001
Arousal composite	0.425 (0.319, 0.521)	<0.0001	−0.344 (−0.455, −0.222)	<0.0001	0.333 (0.225, 0.434)	<0.0001	−0.300 (−0.422, −0.166)	<0.0001
Self-report analyses	(*n* = 320, df = 318)		(*n* = 268, df = 266)		(*n* = 353, df = 351)		(*n* = 235, df = 233)	
Minimum arousal	0.648 (0.579, 0.707)	<0.0001	−0.542 (−0.622, −0.452)	<0.0001	0.602 (0.530, 0.664)	<0.0001	−0.499 (−0.590, −0.397)	<0.0001
Absolute difference	−0.637 (−0.698, −0.567)	<0.0001	0.594 (0.511, 0.666)	<0.0001	−0.636 (−0.695, −0.570)	<0.0001	0.480 ( 0.375, 0.573)	<0.0001
Arousal composite	0.645 (0.576, 0.705)	<0.0001	−0.583 (−0.657, −0.498)	<0.0001	0.624 (0.555, 0.683)	<0.0001	−0.506 (−0.600, −0.404)	<0.0001

To demonstrate a curvilinear or U-shaped relationship, correlations for each break point must have opposite signs and both must be statistically significant.

We conducted additional analyses to examine the degree to which our results depended on data analytic decisions. At least two such decisions for Table 1 could have influenced our results even though we had scientific justification for making those decisions and have consistently made them in past research: analyzing standardized rather than unstandardized arousal data and excluding participants with low genital responses. Neither of these decisions was required to test our hypotheses, however, and some other researchers have not made them (e.g., ref. 22). Seemingly innocuous decisions such as these can hide a lack of robustness of results had other analytic paths been taken (23).

One way to explore the robustness of results across different data analytic decisions is to conduct “multiverse analyses” in which data are analyzed with respect to all combinations of relevant decisions (24). In our case, this required three additional sets of analyses. Each used the two-lines approach, but each used different data: unstandardized arousal data for men who met our inclusion criteria for sexual response; standardized arousal data for all men regardless of degree of response; or unstandardized arousal data for all men regardless of degree of response. Each set of analyses was conducted for each of the dependent variables: Minimum Arousal, Absolute Arousal Difference, and Bisexual Arousal Composite. Furthermore, each analysis was conducted for both break points (i.e., 2.5 and 3.5), and tests with unstandardized data were repeated for the analyses of self-reported arousal. Because each analysis yielded two separate tests (for points left of the break point and for points right of it), this resulted in a total of 48 tests.

*SI Appendix*, Table S1 provides the results for these multiverse analyses. All results were in the direction consistent with increased bisexual arousal for more bisexual Kinsey scores. *SI Appendix*, Fig. S1 also presents the frequency distribution of the 36 exact probabilities for the additional analyses of genital data. Only one *P* value, 0.0503, exceeded the conventional statistical significance threshold, and most of the other 35 *P* values were much smaller. Results for the analyses of self-reported arousal were also consistent, with all *P* values less than 10^−8^. Thus, our general findings persisted regardless of the data analytic decisions we reconsidered.

**Fig. 4.**
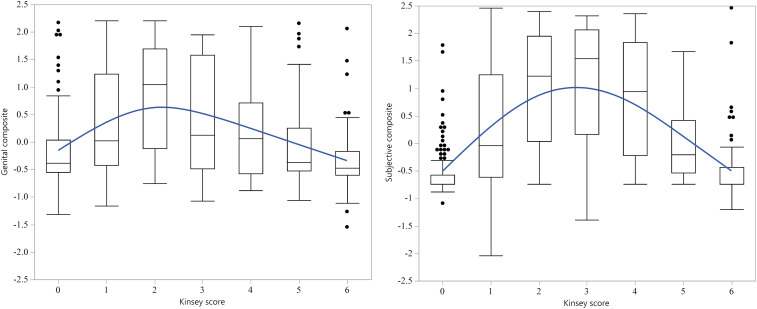
Box plots for the mean standardized genital (*Left*) and self-report (*Right*) Bisexual Composites for men of different Kinsey scores, and a curved line of fit in blue showing the U-shaped trend. The *y* axis is measured in units of within-subjects z-scores. The center line of the box plots represents the median value; the box limits represent the upper and lower quartiles; the whiskers represent the 1.5× interquartile range; individual points represent outliers. Values for the Composites should show an inverted U-shaped distribution if men with Kinsey scores in the bisexual range show bisexual arousal patterns, and a flat distribution if they do not.

Which Kinsey score was associated with the greatest degree of bisexual arousal? To answer this question, we focused on the standardized genital and self-report arousal composites, which correlated *r*(470) = 0.507, 95% CI (0.437, 0.572), *P* < 0.0001. Fig. 4 shows the mean genital and self-report bisexual composites for all Kinsey scores. Higher scores represent greater bisexuality. With respect to the genital composite, Kinsey 2’s showed the strongest evidence for bisexual arousal patterns. With respect to the self-report composite, Kinsey 3′s provided the most bisexual responses. Notably, both contrasts increased steadily to the maximum and then decreased steadily, consistent with a gradation model of sexual orientation.

How bisexual were the arousal patterns of men with bisexual Kinsey scores, compared with other men? It is possible, for example, that bisexual men’s sexual responses are only slightly (albeit statistically significantly) more bisexual than the responses of monosexual men. Or alternatively, the two groups could differ substantially. Answering this question requires a direct comparison of magnitudes of indicators of bisexual response. Two of the main dependent variables we have examined—Minimum Arousal and Absolute Arousal Difference—could be especially informative. Ratios of their means comparing men with bisexual Kinsey scores to men with monosexual scores could helpfully express the answer. To be meaningfully interpreted, ratios require ratio-level measurement, with a true value of zero and interval scaling (25). For example, six inches is twice the length of three inches, but a rating of six on a seven-point Likert scale of current happiness is not meaningfully interpreted as twice a rating of three. Because the data we have primarily focused on so far have been standardized within subjects, it is unsuited to provide meaningful ratios for two reasons. First, the standardized data do not have true zeros, with zero indicating an absence of a quantity. More importantly, standardizing within subjects induces a nonlinear between-subjects transformation of the raw scores, and so the ipsatized data do not have interval-level measurement.

**Fig. 5.**
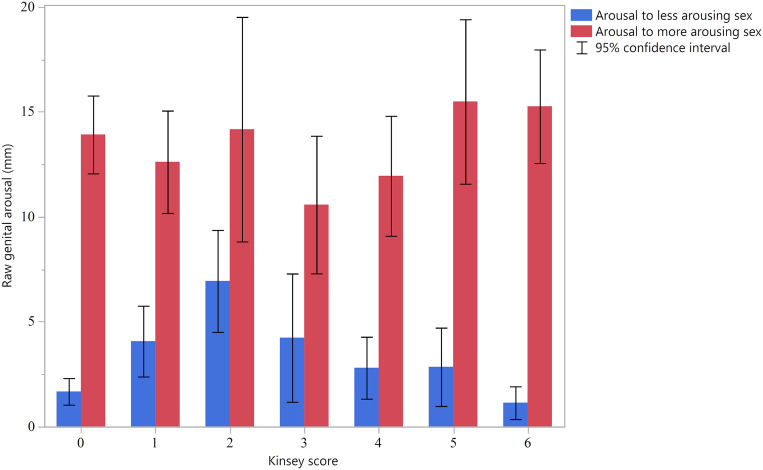
Mean raw (unstandardized) genital response to the more and less arousing sex after subtracting response to neutral stimuli (±95% CI) for men of different Kinsey scores. Units are in millimeters and denote increases in the circumference of the penis. Values for arousal to the less arousing sex should show an inverted U-shaped distribution if men with Kinsey scores in the bisexual range show bisexual arousal patterns, and a flat distribution if they do not.

Fortunately, the raw genital arousal data have a ratio scale, and so we focus on these data for our final analyses. Fig. 5 presents men’s raw genital responses to their more and to their less arousing sex, by Kinsey score. The figure demonstrates that increased bisexuality toward the middle of the Kinsey range is primarily due to increased responding to the less arousing sex. (Neither a two-lines analysis nor a quadratic regression reveals significant evidence for an inverted U effect for the more arousing sex.) Kinsey scores of 0 and 6 were associated with especially low (though not zero) responding to the less arousing sex, which was one of our main indicators of bisexual response. Men with Kinsey scores in the bisexual range (i.e., 2 to 4) produced 3.30 times more response to their less arousing sex compared with the (unweighted) average of men with monosexual Kinsey scores (i.e., 0 and 6). The difference between responses to the more and less arousing sex should be smaller for men with more bisexual Kinsey scores if those scores reflect men’s sexual orientations. Consistent with this prediction, men with Kinsey scores in the bisexual range produced an average difference that was 0.59 times the difference of men with monosexual scores. Both ratios were markedly different from 1. Still, men with Kinsey scores in the bisexual range produced, on average, penile circumference changes that were notably larger to one sex than to the other. The ratio of bisexual men’s genital arousal to their more arousing sex to genital arousal to their less arousing sex averaged 2.62; for monosexual men, it was 10.13. Note that these numbers comprise the ratio of each group’s mean arousal to the more arousing sex divided by their mean arousal to the less arousing sex. They are not the averages of each individual men’s ratios. Some individual ratios are extreme because the denominator is near zero.

In general, results suggested that bisexual men’s arousal patterns were markedly more bisexual than monosexual men’s, and that bisexual men were typically more aroused by one sex than by the other. The combination of our results and the fact that male sexual orientation is bimodally distributed (21) suggests that men with similar high degrees of sexual arousal to both men and women may be especially uncommon.

## Discussion

The primary question motivating this research is whether men who identify as bisexual have sexual arousal patterns that are also relatively bisexual. Results strongly confirmed that men who report attraction to both sexes are more genitally and subjectively aroused by both sexes compared with men who report that they are attracted only to one sex.

The highly consistent evidence for bisexual arousal and orientation from the present study contrasts with inconsistent findings of the past (e.g., ref. 11 [not finding bisexual arousal] and ref. 12 [finding bisexual arousal]). For example, applying the two-lines methodology to the eight individual studies and focusing on the ipsatized genital Bisexual Arousal Composite yielded 29 relevant correlations (i.e., correlations for values on one side of either a 2.5 or 3.5 Kinsey break point, which should be statistically significant for a successful test). Only 12 of these were statistically significant, the median probability equal to 0.073. (*SI Appendix*, Table S2). The comparison of the inconsistent study-level results with the robust results using combined data from all studies demonstrates the increased statistical power of the latter approach.

A second factor that may have contributed to inconsistent results across individual studies is systematic differences between samples of bisexual men. Men who describe themselves as bisexual likely comprise a diverse set of men, some of whom have a bisexual arousal pattern and others who do not. Examples of the latter likely include transitional bisexual men (5, 6) and some paraphilic men who have sexual fantasies involving men but who are not sexually attracted to them (26). Past studies that did not show correspondence between bisexual self-report and bisexual genital arousal had far fewer subjects than the present analyses, and some may have included a higher proportion of men whose bisexual identification was due to reasons other than bisexual arousal. For example, it is possible that the sample of Rieger et al. (11) contained a higher proportion of transitional bisexual men than other samples. Recruitment of participants for that study included advertisements in both alternative and gay-oriented publications, and the bisexual-identified participants may have responded to the advertisement in the gay-oriented publications.

The present research represents the most systematic and extensive assessment of bisexual men’s arousal patterns to date. The data we analyzed comprise all relevant data that the coauthors had collected as of January 2019, and nearly all relevant data of which we are aware. Although we were unable to obtain data from two other studies with relevant data, their inclusion would not have altered our general conclusions even if we assume that those subsamples would not have shown significant bisexual arousal patterns (*SI Appendix*, *Supplementary Text*).

The primary limitation of this study is that participants were necessarily volunteers. Thus, the degree to which they are representative of men across the Kinsey scale is unknown. This limits confident generalization about the magnitude of our results. However, it is unclear how the basic pattern of results—greater bisexual response for men with more bisexual Kinsey scores—could be entirely an artifact of volunteer bias. Additionally, the fact that participants were volunteers sampled exclusively from a few Western countries prevents us from knowing how general the patterns we have observed are. However, we are unaware of promising theories specifying how these patterns might vary cross-culturally.

In a recent highly publicized article on genetic determinants of same-sex versus opposite-sex sex partners, there was no clear genetic gradient distinguishing persons with a high proportion of same-sex partners from those with opposite-sex partners (27). The authors asserted that, because of their negative findings, the validity of the Kinsey scale should be reconsidered. Our findings support the opposite conclusion, and we believe they are more relevant with respect to the validity of self-reported sexual orientations. When we ask men to assess themselves on the Kinsey scale, we do not mean for them to guess their underlying genotypes. Rather, we are asking them about their relative sexual feelings for women and men. Sexual arousal patterns are closely related to these feelings in men; indeed, they are detectable and likely lead to the subjective experience of attraction and desire (1). We have demonstrated that both genital and self-reported sexual arousal to male and female erotic stimuli form a gradient over the Kinsey scale, regardless of their underlying causes.

## Materials and Methods

### Participants.

Participants comprised those of available studies known to us that included genital measures of sexual arousal in men who also reported their Kinsey scores, with four exceptions. Two studies focused on men with paraphilias (26, 28), and those data were intentionally excluded. Two other studies containing relevant data could not be included because the authors did not respond to our requests for data (14, 23). The unavailable studies comprised genital assessment data of a total of 89 men, including 23 who identified as bisexual.

Participants for the constituent studies were recruited by researchers at four sites: Northwestern University in Evanston, IL (6, 11, 12, 29), the Centre for Addiction and Mental Health in Toronto, Ontario, Canada (18), the University of Essex in Colchester, UK (15, 30), and Cornell University in Ithaca, NY (17). Individual sample sizes and methodological differences between the studies are reported in Table 2.

**Table 2.** Comparison of data sources

Study	*N*	Location	Orientation wording	Mean age (SD)	Stimuli type	Stimuli duration	Hardware
Chivers et al. (18)	45	Toronto, Canada	Attraction	24.58 (4.83)	Partnered sexual activity	90 s	Limestone
Jabbour et al. (29)	96	Evanston, IL	Attraction	30.00 (9.35)	Partnered sexual activity	3 min	MP150
Rieger et al. (11)	101	Evanston, IL	Fantasies	31.13 (6.01)	Partnered sexual activity	2 min	MP100
Rieger et al. (17)	76	Ithaca, NY	Attraction	24.38 (6.53)	Solo masturbation	3 min	MP100
Rosenthal et al. (12)	102	Evanston, IL	Fantasies	34.73 (7.31)	Partnered sexual activity	3 min	MP100
Semon et al. (6)	36	Evanston, IL	Attraction	26.92 (6.13)	Partnered sexual activity	3 min	MP150
Slettevold et al. (15)	109	Colchester, UK	Attraction	23.80 (9.42)	Solo masturbation	3 min	MP150
Watts et al. (30)	41	Colchester, UK	Attraction	30.85 (13.41)	Solo masturbation	3 min	MP150

Sample sizes presented in this table do not include participants who were excluded from our analyses for genital low response, poor data quality, or missing data.

Across the constituent studies, data for 606 participants were available. All participants were cisgender (i.e., no participants were transgender). Of these, 474 participants were included in our main genital arousal analyses. Of the 132 excluded participants, 96 participants were excluded for exhibiting insufficient genital arousal for meaningful analysis. In any given study of male sexual arousal, there is a proportion of low-responding participants who do not become substantially aroused to any of the stimuli (among the constituent studies, this proportion ranges between 4.95% and 26.73%): Typical self-reported reasons for low response include discomfort and disinterest in the actors or actions featured in the stimuli. We counted as low responders (and excluded from initial analyses) participants who either 1) did not exhibit an average change of at least 2 mm in penile circumference to male or female stimuli compared to a baseline value; or 2) did not produce standardized mean genital arousal to at least one erotic stimulus category that exceeded that to neutral stimuli by more than half of an SD. These criteria have been used in most of the studies included herein (6, 11, 12, 15, 17, 29). Another 36 participants were excluded from genital analyses because their data were incomplete or of poor quality (e.g., impossible values because of technical difficulties when running those participants). Regarding the self-report analyses, 12 participants were excluded from self-reported arousal analyses due to not providing self-reported data, and an additional six participants were excluded for reporting arousal scores of 0 for all stimuli. This resulted in a sample size of 588 men for self-report analyses.

Within the total sample of 606 men, 178 participants self-identified as exclusively heterosexual, 102 identified as mostly heterosexual, 46 as bisexual leaning heterosexual, 34 as bisexual, 37 as bisexual leaning homosexual, 70 as mostly homosexual, and 139 as exclusively homosexual. Note that this distribution of sexual identities is not representative of the overall population. Homosexual- and bisexual-identified men were over-sampled because the focus of the component studies was typically on sexual orientation variation. This nonrepresentative sampling increased statistical power to detect differences in arousal patterns in different regions of the Kinsey scale. The average age was 28.63 y (SD = 9.03). Data for educational attainment were available for 359 participants and were coded as 1 (no high school), 2 (some high school), 3 (high school diploma), 4 (some college), 5 (college graduate), and 6 (postgraduate student or degree). The average level of educational attainment was 4.76 (SD = 0.85), and the most common response was “college graduate” (*n* = 133). Data for ethnicity were available for 502 participants. Of these, 326 (64.94%) were White/Caucasian, 60 (11.95%) were Black, 42 (8.37%) were Asian, 29 (5.78%) were Hispanic/Latino, and 45 (8.96%) reported other. Distributions of age, ethnicity, and educational attainment by sexual orientation are reported in Table 3.

**Table 3.** Participant demographics by self-reported sexual orientation

	Exclusively heterosexual	Mostly heterosexual	Bisexual leaning heterosexual	Bisexual	Bisexual leaning homosexual	Mostly homosexual	Exclusively homosexual
Kinsey score	0	1	2	3	4	5	6
*N*	178	102	46	34	37	70	139
*N* after all exclusions	138	80	35	26	33	51	107
Mean age (SD)	27.66 (9.05)	29.62 (8.52)	31.33 (10.25)	29.00 (9.99)	29.81 (8.93)	27.28 (6.80)	28.51 (9.53)
Mean education (SD)	4.63 (0.81)	4.76 (0.76)	4.88 (0.91)	4.68 (1.04)	4.87 (1.02)	4.86 (0.86)	4.77 (0.81)
Percentage non-White	40.76	28.41	24.24	46.15	54.17	27.78	32.50

Education data were available for 359 participants; educational attainment was not recorded in Jabbour et al. (29), Watts et al. (30), or Slettevold et al. (15). Education was coded as 1 (no high school), 2 (some high school), 3 (high school diploma), 4 (some college), 5 (college graduate), or 6 (postgraduate student or degree). Ethnicity data were available for 502 participants; ethnicity was not recorded in Rosenthal et al. (12).

### Measures.

#### Sexual orientation.

Participants reported their sexual orientation using the seven-point Kinsey scale (4) ranging from 0 (exclusive heterosexual orientation) to 6 (exclusive homosexual orientation), with 3 representing bisexual orientation with equal attraction to men and women. In most studies, the prompt for the scale was worded such that it framed sexual orientation as one’s relative attraction to men versus women. However, two of the included studies (11, 12) (*n* = 203, or 33% of the overall sample) framed sexual orientation as one’s relative frequency of sexually fantasizing about men versus women.

#### Genital arousal.

Each study assessed changes in the penile circumference of participants when viewing erotic stimuli, with increases in circumference denoting increased genital arousal (31). The majority of the data were collected using an indium/gallium strain gauge connected to either an MP150 or an MP100 data acquisition unit alongside AcqKnowledge software. Data from Rieger et al. (11) were collected using a mercury-in-rubber strain gauge. Chivers et al. (18) used the Limestone hardware and software and a mercury-in-rubber strain gauge.

#### Subjective arousal.

Participants subjectively reported their arousal to male and female erotic stimuli and to neutral stimuli following each stimulus. The particular range of each study’s subjective arousal measure varied (e.g., an 11-point scale was used in Jabbour et al. (29) whereas a seven-point scale was used in Rieger et al. (11)). Thus, all subjective ratings for arousal to male stimuli and arousal to female stimuli were rescaled as proportions of the maximum possible response.

### Procedure.

In each constituent study, participants privately viewed various erotic video clips while a penile strain gauge was used to measure changes in the circumference of the penis. Most of the studies utilized 3-min clips; Rieger et al. (11) used 2-min clips, and Chivers et al. (18) used 90-s clips. Neutral stimuli (e.g., footage of landscapes and wildlife) were included in each paradigm to assess a baseline level of arousal. Erotic stimuli were presented in random order; these included either a male stimulus (depending on the study, either male–male sexual acts or one male masturbating) or female stimulus (female–female sexual acts or one female masturbating). During or after each stimulus, participants provided a subjective arousal rating. If participants were still aroused before the presentation of the next sexual stimulus (e.g., if their penile circumference exceeded the previously assessed baseline by 2 mm), they were instructed via intercom to perform a distracting task (e.g., “in your head, count all of the multiples of 9”) until they returned to their baseline level and the next stimulus began. After each session, participants were debriefed and compensated for their time.

### Data Analysis.

Each individual’s raw genital responses to appropriate stimuli were averaged to provide three values: average arousal (i.e., penile circumference) to neutral stimuli, to male stimuli, and to female stimuli. Raw genital measures were in units of millimeters. Analogously, self-reported ratings were averaged to provide the same three values, in units of proportion of maximum possible ratings. These values were used to produce all subsequent metrics.

For the main analyses, genital and self-reported arousal scores were standardized within participants, using each participant’s average arousal scores for male, female, and neutral erotic stimuli. This practice, also called ipsatizing, is useful to remove unwanted sources of variation, including those attributable to penis size and general responsiveness (32). Each man’s standardized arousal to male and to female stimuli was then transformed by subtracting arousal to neutral stimuli.

The primary analyses in Table 1 comprised a version of the two-lines test (16). The rationale of the test is that, if the relation between two variables is U-shaped (or inverted U-shaped), there must be a point on the predictor range, x_C_, such that the regression line using values below x_C_ has an opposite sign of the regression line using values above x_C_. Our analysis diverged from that outlined by Simonsohn in two ways. First, we presented Pearson correlations rather than unstandardized regression coefficients to make it easier for the reader to assess the magnitude of line slopes. Second, instead of allowing Simonsohn’s algorithm to find the ideal break point, x_C_, we present results for two different break points, one on either side of the midpoint of the Kinsey scale. (One must not use a value for x_C_ that exists in the data, and thus 3 could not be used.) This meant that, for both tests, the middle of the Kinsey scale provided the most bisexual scores on the dependent variables as well as an examination of the robustness of results.

## Supplementary Material

Supplementary File

## Data Availability

All data analyzed in the present study are available at the Open Science Framework website at the following web address: https://osf.io/qcmgp/. Formulae used to derive the variables used in the present study are also available at said web address, both in the provided data files and a Word document labeled “Explanation of variables in the data file” that has been deposited alongside the data.
