# Supplementary material for: Robust evidence for bisexual orientation among men
Source: Proc Natl Acad Sci U S A. 2020 Jul 20;117(31):18369–77. doi: 10.1073/pnas.2003631117 (PMC7414168; doi:10.1073/pnas.2003631117)
Supplement: Supplementary File [file pnas.2003631117.sapp.pdf]

PNAS
www.pnas.org

Supplementary Information for

# Robust Evidence for Bisexual Orientation Among Men

Jeremy Jabbour, Luke Holmes, David Sylva, Kevin J. Hsu, Theodore L. Semon, Alan M. Rosenthal, Adam Safron, Erlend Slettevold, Tuesday M. Watts-Overall, Ritch C. Savin-Williams, John Sylla, Gerulf Rieger, John Michael Bailey

Address correspondence to J. Michael Bailey
Email: jm-bailey@northwestern.edu.

**This PDF file includes:**

Supplementary text
Figure S1
Tables S1 to S2
SI References

www.pnas.org/cgi/doi/10.1073/pnas.2003631117

**Supplementary Text.**

Data from two studies were unavailable to us (1, 2). We examined whether our results would hold if we simulated the data from the missing studies under the most ungenerous assumptions plausible, which is that they would not show a bisexual arousal pattern in bisexual-identified men. We focused on the ipsatized genital Bisexual Composite.

Tollison et al. (1) did not provide Kinsey scores, and so we analyzed the data from all studies using a three-level predictor: Heterosexual, Bisexual, and Homosexual. The two missing studies included a total of 37 heterosexual, 23 bisexual, and 29 homosexual men. In order to check how our results would change if those studies' data were unfavorable, we assigned data values for each of these men as follows: For the heterosexual and homosexual men, we assigned the value of the dependent variable that was the mean of their respective groups: 0.018 and -0.240. For the bisexual men, we assigned the value of the dependent variable that was the average of those two values: -0.111. (Note that for our sample, the actual mean for bisexual men was 0.500.) Thus, we biased our analysis against showing differences between bisexual men and both homosexual and heterosexual men.

We conducted analyses for the dataset that included both our actual data and the simulated data from the previously omitted studies. The analogous analyses to our two-lines approach compared the difference between heterosexual and bisexual men and then the difference between bisexual and homosexual men. The mean ipsatized genital Bisexual Composite for bisexual men, M = 0.380 (SD = 0.913) significantly exceeded that for heterosexual men, M = 0.018 (SD = 0.774), $t$ (370) = 3.952, $p < .0001$; it also exceeded the mean for homosexual men, M = -0.244 (SD = 0.573), $t$ (306) = 7.366, $p < .0001$.

Thus, our results remain highly significant even if we simulate data from the missing studies that are as opposed to our main findings as they plausibly could be.

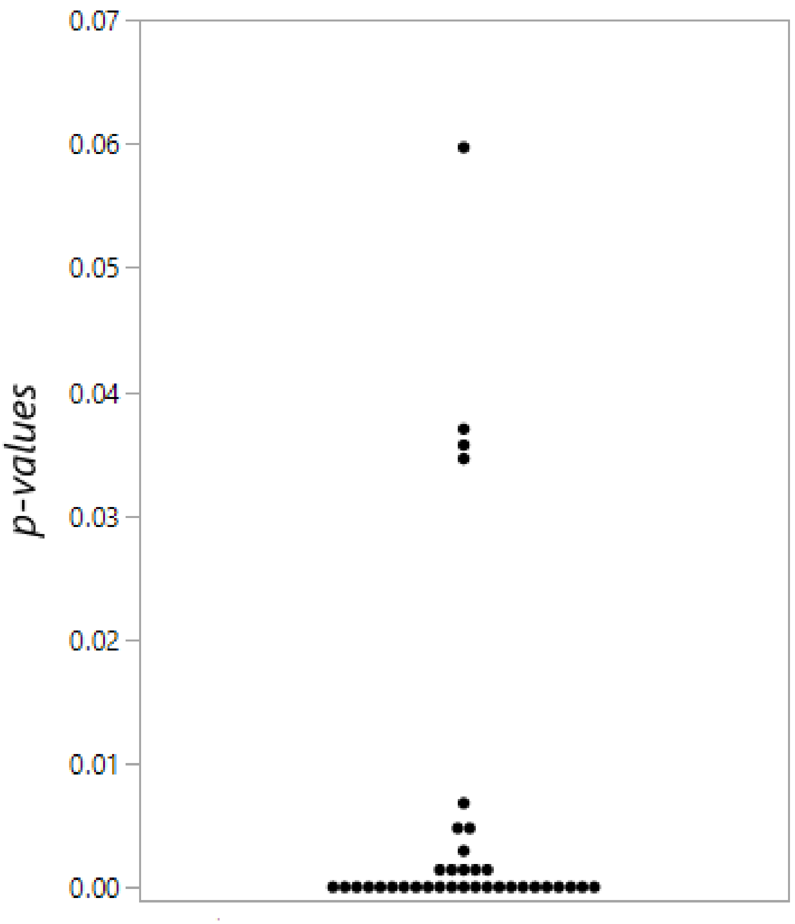


***Fig. S1***. Frequency distribution of exact probabilities from tests of genital data in the multiverse analysis.

Table S1.

*p-values of two-lines analyses for multiverse analyses*

| Breakpoint | 2.5 | | | | | | 3.5 | | | | | |
|---|---|---|---|---|---|---|---|---|---|---|---|---|
| | Minimum arousal Line | | Absolute difference Line | | Composite variable Line | | Minimum arousal Line | | Absolute difference Line | | Composite variable Line | |
| | Below Break | Above Break | Below Break | Above Break | Below Break | Above Break | Below Break | Above Break | Below Break | Above Break | Below Break | Above Break |
| **Genital arousal** | | | | | | | | | | | | |
| Unstandardized with exclusions | 4.86e-7 | 4.46e-3 | 2.99e-3 | 3.39e-4 | 6.32e-8 | 9.05e-7 | 6.04e-5 | 0.0370 | 4.57e-4 | 0.0346 | 4.26e-7 | 1.05e-3 |
| Including low-responders, standardized | 3.79e-9 | 1.23e-4 | 1.02e-8 | 1.54e-6 | 1.01e-10 | 2.68e-6 | 3.79e-7 | 2.09e-3 | 8.93e-7 | 2.31e-4 | 3.57e-8 | 2.07e-4 |
| Including low-responders, unstandardized | 6.96e-7 | 6.26e-3 | 1.65e-3 | 6.61e-4 | 1.05e-8 | 1.45e-6 | 3.16e-5 | 0.0413 | 3.87e-4 | **0.0503** | 6.58e-8 | 1.52e-3 |
| **Self-report** | | | | | | | | | | | | |
| Unstandardized with exclusions | 2.07e-29 | 3.42e-25 | 1.74e-16 | 2.44e-19 | 1.45e-33 | 4.89e-30 | 1.77e-38 | 1.36e-13 | 4.57e-22 | 1.13e-8 | 2.13e-44 | 1.60e-14 |

To demonstrate a curvilinear or U-shaped relationship, correlations on opposite sides of the dashed lines must have opposite signs and both must be statistically significant. All corresponding correlations had opposite signs, so for the sake of increased readability, we report only exact *p*-values here. *p*-values that are no longer statistically significant following a multiverse analysis are bolded. All genital analyses exclude participants flagged as having poor or missing genital data.

Table S2.

*Results of two-lines analyses for the genital bisexual composite across individual study samples, for both breakpoints.*

| Breakpoint | 2.5 | | | | | | 3.5 | | | | | |
|---|---|---|---|---|---|---|---|---|---|---|---|---|
| | Slope for Kinsey 0-2 | | | Slope for Kinsey 3-6 | | | Slope for Kinsey 0-3 | | | Slope for Kinsey 4-6 | | |
| | *N* | *r* | *p* | *N* | *r* | *p* | *N* | *r* | *p* | *N* | *r* | *p* |
| Chivers et al. (3) | 22 | .289 | **.192** | 16 | .053 | **.845** | 22 | .289 | **.192** | 16 | .053 | **.845** |
| Jabbour et al. (4) | 78 | .381 | <.001 | 0 | - | - | 78 | .381 | <.001 | 0 | - | - |
| Rieger et al. (5) | 31 | .306 | **.094** | 37 | .065 | **.702** | 36 | .055 | **.749** | 32 | .021 | **.909** |
| Rieger et al. (6) | 29 | .500 | .006 | 34 | -.192 | **.278** | 33 | .264 | **.137** | 30 | -.359 | **.051** |
| Rosenthal et al. (7) | 38 | .471 | .003 | 49 | -.634 | <.001 | 46 | .464 | .001 | 41 | -.451 | .003 |
| Semon et al. (8) | 0 | - | - | 31 | -.539 | .002 | 0 | - | - | 31 | -.539 | .002 |
| Slettevold et al. (9) | 49 | .542 | <.001 | 41 | -.307 | **.051** | 58 | .416 | .001 | 32 | -.370 | .037 |
| Watts et al. (10) | 6 | -0.43 | **.400** | 13 | .171 | **.578** | 6 | -.426 | **.400** | 13 | .170 | **.578** |

To demonstrate a curvilinear or U-shaped relationship, correlations on opposite sides of the dashed lines must have opposite signs and both must be statistically significant. *p*-values that are not statistically significant are bolded.

## SI References


1. C. D. Tollison, H. E. Adams, J. W. Tollison, Cognitive and physiological indices of sexual arousal in homosexual, bisexual, and heterosexual males. *Journal of Behavioral Assessment*. **1**, 305-314 (1979).
2. J. A. Cerny, E. Janssen, Patterns of sexual arousal in homosexual, bisexual, and heterosexual men. *Archives of Sexual Behavior*. **40**, 687-697 (2011).
3. M. L. Chivers, M. C. Seto, R. Blanchard, Gender and sexual orientation differences in sexual response to sexual activities versus gender of actors in sexual films. *Journal of Personality and Social Psychology*. **93**, 1108-1121 (2007).
4. J. Jabbour, K. Hsu, J. M. Bailey, Sexual arousal patterns of mostly heterosexual men. *Archives of Sexual Behavior.* Advanced online publication. https://doi.org/10.1007/s10508-020-01720-z. Accessed 28 May, 2020.
5. G. Rieger, M. L. Chivers, J. M. Bailey, Sexual arousal patterns of bisexual men. *Psychological Science*. **16**, 579-584 (2005).
6. G. Rieger et al., Sexual arousal: The correspondence of eyes and genitals. *Biological Psychology*. **104**, 56-64 (2015).
7. A. M. Rosenthal, D. Sylva, A. Safron, J. M. Bailey, The male bisexuality debate revisited: Some bisexual men have bisexual arousal patterns. *Archives of Sexual Behavior*. **41**, 135–147 (2012).
8. T. L. Semon, K. J. Hsu, A. M. Rosenthal, J. M. Bailey, Bisexual phenomena among gay-identified men. *Archives of Sexual Behavior*. **46(1)**, 237-245 (2017).
9. E. Slettevold et al., Bisexual men with bisexual and monosexual genital arousal patterns. *Biological Psychology*. **148**, 107763 (2019).
10. T. M. Watts, L. Holmes, J. Raines, S. Orbell, G. Rieger, G. (2018). Sexual arousal patterns of identical twins with discordant sexual orientations. *Scientific Reports.* **8(1)**, 1-7 (2018).
